# TRACES: A Lightweight Browser for Liquid Chromatography–Multiple Reaction Monitoring–Mass Spectrometry Chromatograms

**DOI:** 10.3390/metabo12040354

**Published:** 2022-04-15

**Authors:** Yoshihiro Kita, Suzumi M. Tokuoka, Yoshiya Oda, Takao Shimizu

**Affiliations:** 1 Life Sciences Core Facility, Graduate School of Medicine, The University of Tokyo, 7-3-1 Hongo, Bunkyo-ku, Tokyo 113-0033, Japan; 2 Department of Lipidomics, Graduate School of Medicine, The University of Tokyo, 7-3-1 Hongo, Bunkyo-ku, Tokyo 113-0033, Japan; stokuoka@m.u-tokyo.ac.jp (S.M.T.); yoda@m.u-tokyo.ac.jp (Y.O.); tshimizu@ri.ncgm.go.jp (T.S.); 3 Department of Lipid Signaling, National Center for Global Health and Medicine, 1-21-1 Toyama, Shinjuku-ku, Tokyo 162-8655, Japan

**Keywords:** LC-MRM-MS, deisotoping, software, targeted lipidomics, targeted metabolomics, phospholipids

## Abstract

In targeted metabolomic analysis using liquid chromatography–multiple reaction monitoring–mass spectrometry (LC-MRM-MS), hundreds of MRMs are performed in a single run, yielding a large dataset containing thousands of chromatographic peaks. Automation tools for processing large MRM datasets have been reported, but a visual review of chromatograms is still critical, as real samples with biological matrices often cause complex chromatographic patterns owing to non-specific, insufficiently separated, isomeric, and isotopic components. Herein, we report the development of new software, TRACES, a lightweight chromatogram browser for MRM-based targeted LC-MS analysis. TRACES provides rapid access to all MRM chromatograms in a dataset, allowing users to start ad hoc data browsing without preparations such as loading compound libraries. As a special function of the software, we implemented a chromatogram-level deisotoping function that facilitates the identification of regions potentially affected by isotopic signals. Using MRM libraries containing precursor and product formulae, the algorithm reveals all possible isotopic interferences in the dataset and generates deisotoped chromatograms. To validate the deisotoping function in real applications, we analyzed mouse tissue phospholipids in which isotopic interference by molecules with different fatty-acyl unsaturation levels is known. TRACES successfully removed isotopic signals within the MRM chromatograms, helping users avoid inappropriate regions for integration.

## 1. Introduction

Liquid chromatography–multiple reaction monitoring–mass spectrometry (LC-MRM-MS) is the most commonly used MS/MS-based method for determining known compounds using triple quadrupole mass spectrometry (TQMS). Compared with scan-based LC-MS methods that utilize quadrupole time-of-flight (Q-TOF) or Fourier transform (FT)-based MS instruments, LC-MRM-MS has advantages in sensitivity with limited target coverage. In the early days, a single MRM data acquisition event required 10–50 ms or more dwell time, which did not allow acquisition methods to accommodate more than 50–100 MRMs to ensure a chromatographically sufficient data sampling rate. Accordingly, LC-MRM-MS has been used to quantify a relatively small number (less than 100) of target compounds [1].

The recent introduction of high-speed TQMS, which can perform more than 500 MRM events/s and ionization polarity switching, in combination with a modern data acquisition software that provides flexible scheduling of MRM events (known as ‘scheduled MRM’) has enabled ‘widely targeted’ LC-MRM-MS analyses, accommodating ~300 or more MRMs in a single analytical run. These performance improvements make proteomics and metabolomics practical when using LC-MRM-MS [2,3].

While data acquisition has greatly changed, a limited number of software packages can handle large-scale LC-MRM-MS datasets. For proteomics, tools such as Skyline [4], mProphet [5], and Ariadne [6] are known for MRM-based proteomics. For metabolomics, MRMPROBS/MRM-DIFF has been reported [7,8], while Skyline has been recently equipped with a small-molecule mode [9]. These software packages provide processing and visualization of target peaks throughout a large dataset and summarize and export numerical data for downstream statistical analysis software packages.

Although existing tools are intended to automate a series of processes and advances in algorithms have greatly improved their performance, human inspection of raw data in detail during development and in the preliminary stages of analysis remains a critical step. Because LC-MRM-MS is a methodology that does not acquire mass spectra, the information available for component identification is limited to the retention time and peak shape of the chromatogram and the chromatograms of other MRM channels acquired at the same time. Real samples with biological matrices often cause complex chromatographic patterns due to non-specific, insufficiently separated, isomeric, and isotopic components, and it is not always easy to draw a clear conclusion from this limited information [10]. Regarding interfering signals on chromatograms caused by isotopologues, several software tools have implemented isotope correction algorithms because the mechanism of their occurrence is clear. For example, LICAR can perform isotope correction of MRM peak area values for 25 predefined lipid classes [11]. However, this software assumes that the peaks are not chromatographically separated and cannot be used with the commonly used reversed-phase chromatography. MRMPROBS/MRM-DIFF allows the user to provide a compositional formula for isotopic correction of peak areas, but the calculations are based on MS1-level isotopic distribution ratios, which lack theoretical validity [7,8]. In addition, isotope correction in existing software packages focuses on the quantitative correction of peak areas and does not aid in the qualitative interpretation of complex chromatogram patterns. Therefore, manual review is necessary, which is a bottleneck in LC-MRM-MS method development, as well as in the early phase of its deployment.

This study aimed to prepare a software tool that facilitates the data-reviewing process of a large-scale LC-MRM-MS dataset with complex chromatographic patterns. The features of the new software, TRACES, and its application to a phospholipid LC-MRM-MS dataset are described.

## 2. Results and Discussion

### 2.1. Software Design Concept and Workflow

**Figure 1:**
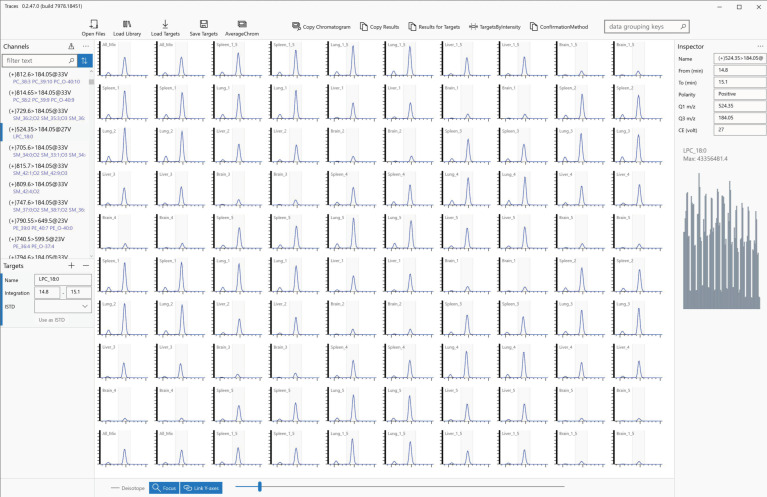
TRACES graphical user interface.

TRACES was developed as stand-alone software and can be installed on a standard Windows 10 PC. Vendor-specific LC-MS data files or vendor-neutral mzML files are converted into TRACES (.trc) binary data files using the TRACES data file converter. Users can start browsing the data by loading them into the software. This simplicity differentiates TRACES from other software that require complex preparation, such as creating projects or defining analysis before loading data. Figure 1 illustrates the graphical user interface of TRACES. When new data files are loaded, TRACES extracts MRM channels and lists them in the ‘Channels’ pane. Users can use a search (filter) box to quickly narrow down the candidate channels of interest, even from more than 1000 channels (see below). In addition, MRM channels can be sorted according to the signals in the data, allowing users to prioritize browsing from channels with signals.

TRACES displays chromatograms for the selected MRM channel for all loaded data files (Figure 1). User operations to chromatograms, such as changing the selection of the integration range and adding retention time anchor points for global retention time alignment (see below), are immediately applied to all the data. In addition, each MRM channel has a default integration range (entire chromatogram) that users can use untouched or modified. Thus, a quick output can be obtained in ad hoc analyses by changing the integration range where necessary.

### 2.2. Retention Time Alignment

**Figure 2:**
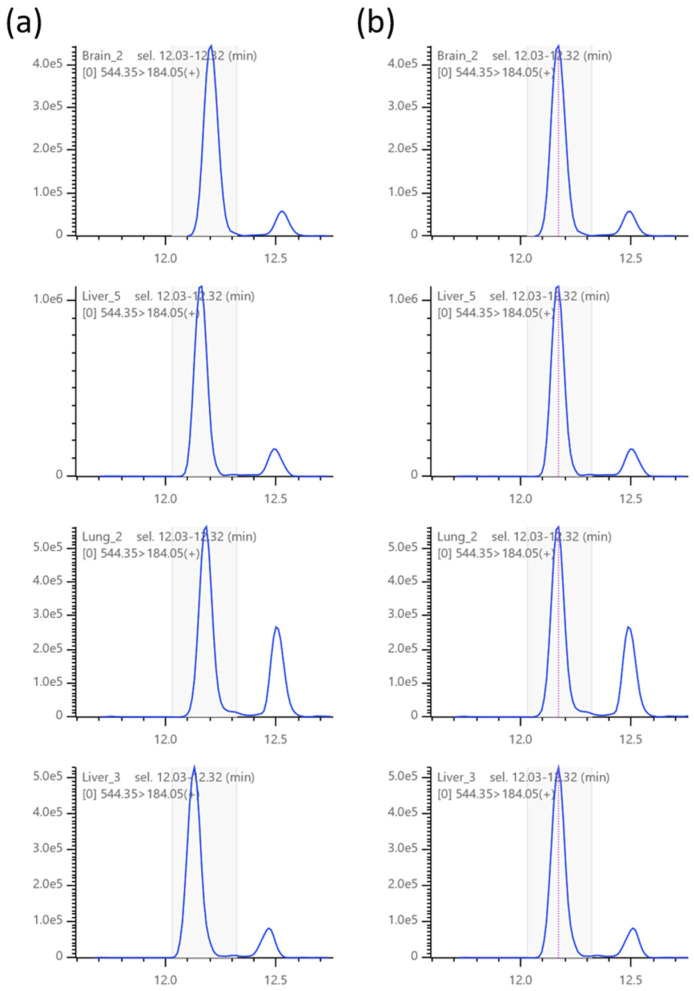
Global chromatogram alignment. Chromatograms before (**a**) and after (**b**) alignment. Red dotted lines indicate the retention time anchor point, which can be added by specifying the region containing the peak of interest and clicking the ‘Add retention time anchor’ button in the Channels pane.

Retention time alignment is one of the most important functions of the software in this category. After removing run-to-run fluctuations in retention time variation, users can visually compare local chromatographic features with high confidence. To this end, TRACES provides software-assisted manual global retention time alignment. Using a simple operation, users can specify any peak as a retention time anchor point. Next, using the anchor points, TRACES transforms the original retention times of the dataset and draws chromatograms using the corrected retention time axis (Figure 2). The current implementation of TRACES does not support an automated retention time alignment because of the assumption that the number of MRM channels varies greatly depending on the user’s experimental design, which prevents algorithm-based automatic selection of reliable peaks for alignment.

### 2.3. Compound Library

**Figure 3:**
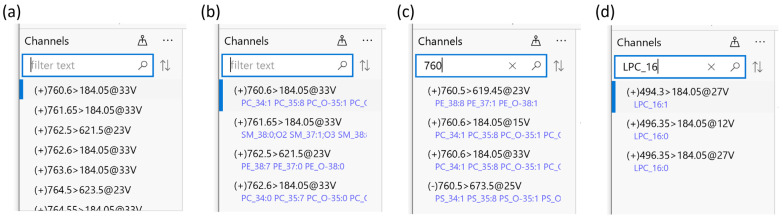
Channels pane before (**a**) and after (**b**) loading the compound library. Compounds matched to the MRM channels are listed below each channel. (**c**,**d**) Channels can be filtered using the search box. By searching ‘760’ (**c**) or ‘LPC_16’ (**d**), users can quickly narrow down channels with *m*/*z* 760 (**c**) or compound names containing ‘LPC_16’ (**d**), respectively.

The compound library is a text file that describes compound information, including formulae and MRM transitions (Table 1). When the library is loaded, TRACES links the compound to the MRM channels of the current dataset (Figure 3a,b). The link does not necessarily imply identification but indicates possible detection targets for the channel. For example, in Figure 3b, the MRM channel ‘(+)760.6 > 184.05@33V’ aimed at phosphatidylcholine (PC) 34:1 has been linked with three additional library hits: PC 35:8, PC O-35:1, and PC O-36:8. In TRACES, measurement and analysis are separated by interpreting the MRM channels at the time of data analysis. Users can use compound names and MRM transition values in the search box to filter channels (Figure 3c,d). The annotations are also used for deisotoping the chromatograms, as described below.

### 2.4. Integration Target

**Figure 4:**
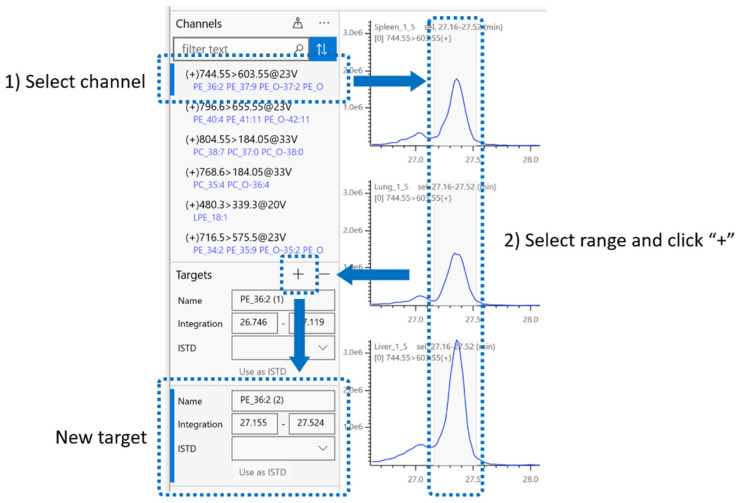
Integration target. Each channel can have one or more integration targets. To create a new target, (1) select a channel and then (2) select integration range and click the ‘+’ button in the ‘Targets’ pane. Channels can be searched by the names of targets they have.

In addition to the default integration range, users can create ‘targets’, which are specified integration ranges. By clicking a specific button during data browsing, a new target is created for the currently selected chromatogram region, which appears in the ‘Targets’ pane (Figure 4). A target is defined simply as an MRM channel and integration range (start and end times) and does not depend on peak detection. Therefore, any chromatogram region of interest that may contain single or multiple peaks or features can be set as a target to calculate the background-subtracted area under the curve. Users may create targets on an ad hoc basis or export them as target definition files (.tdf) for reuse. 

### 2.5. Isotopic Correction of Chromatograms

MRM chromatograms of biological samples often contain off-target signals. Since MRM filters the target compound by precursor and product ion *m*/*z*, any other compounds that match the conditions will cause signals. Such compounds include isomeric and isobaric compounds with similar fragmentation patterns. For these, TRACES supports the interpretation process by providing hints through compound library-based channel annotation, as mentioned above.

**Figure 5:**
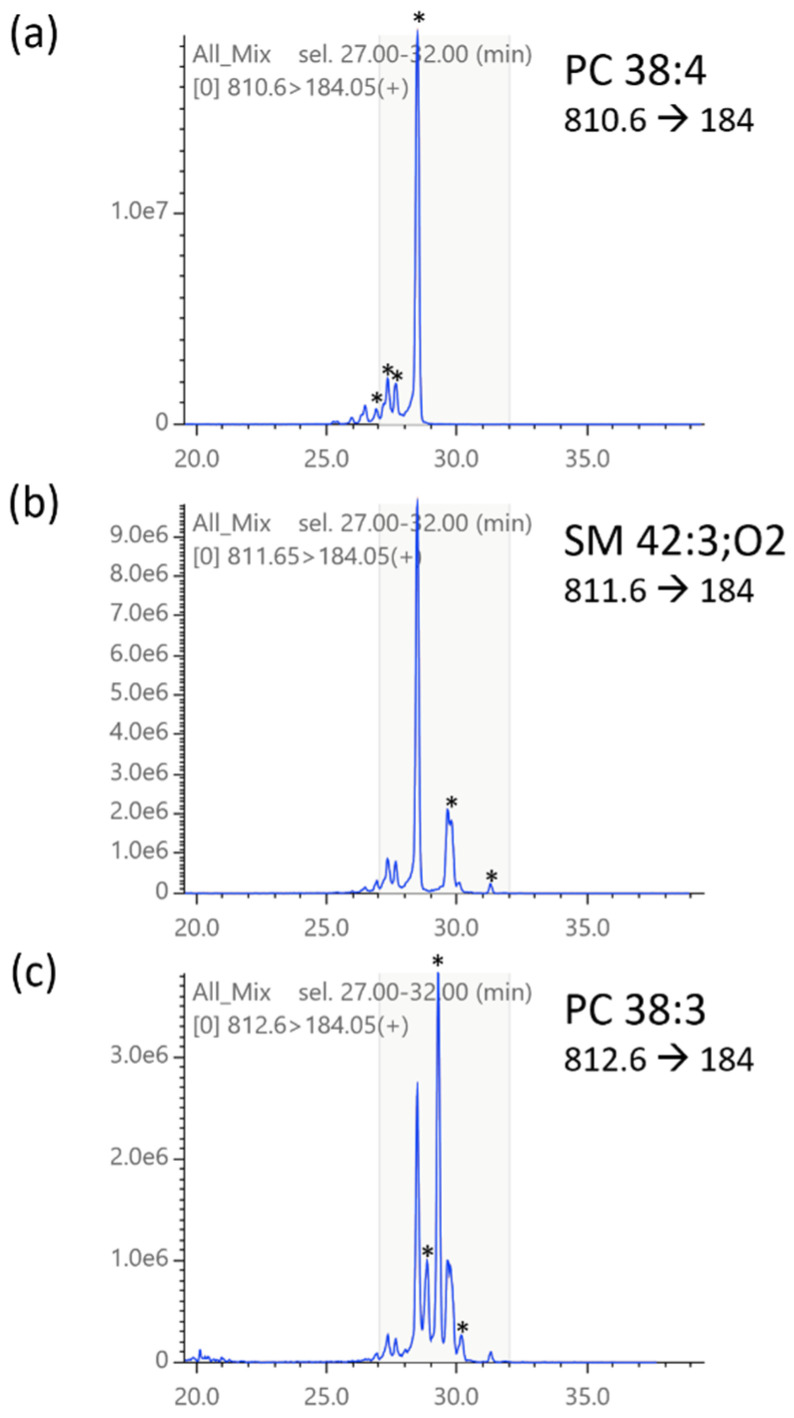
Isotopic interference of MRM chromatograms. A mixture of mouse tissue lipid extract (spleen, lung, liver, and brain) was analyzed. In each chromatogram, asterisks indicate possible monoisotopic peaks. In the middle panel (targeting SM 42:3;O2), interfering signals from M1 isotopologues of putative PC 38:4 are obvious. In the bottom panel (**c**), interfering signals from M1 isotopologues of putative SM 42:3;O2 (**b**) as well as M2 isotopologues of putative PC 38:4 (**a**) are observed.

Isotopic signals are often problematic, a well-known issue in lipidomics [10,11,12,13,14]. As shown in Figure 5, the isotopic signals of a phospholipid with a lower precursor *m*/*z* appear within the MRM chromatograms of those with a higher precursor *m*/*z*. Depending on the chromatographic separation, they either appear at different retention times as the target peaks or partially or fully overlap with them. There is generally a many-to-many relationship between the compound causing interference and the affected MRM chromatograms, complicating the problem. We must also consider nested relations such that the target compound of a chromatogram receiving interference affects other chromatograms. Thus, locating the true signal of a target compound on a chromatogram and confirming its purity require validation by referencing related chromatograms with different MRM transitions, a time-consuming and skill-demanding task. Notably, isotopic correction of MRM signals requires the calculation of MS2-level isotopic distributions, which have not been supported or partially implemented in existing software packages. For example, isotopic correction is not implemented in Skyline [4,9]. This may be because it handles chromatograms of multiply charged peptides (i.e., the *m*/*z* interval of isotopologues is smaller than the resolution of TQMS and therefore not separated). MRMPROBS/MRM-DIFF [7,8] performs isotopic correction of peak areas but uses MS1-level isotopic distribution; therefore, the results are invalid. Very recently, LICAR has been reported to perform proper isotopic correction for peak areas [11], but it is assumed that isotopic peaks completely overlap with the target peak. Therefore, its application is limited to chromatography with lipid class-based separation (e.g., hydrophilic interaction chromatography or supercritical fluid chromatography) or direct-infusion/flow injection analysis. 

Table 2 and Table 3 show the MS1 and MS2 isotopic distributions of PC 34:2 and phosphatidylserine (PS) 34:2, respectively. As the number of isotopes in the precursor molecule increases, the number of product ion isotopologues also increases. The *n*-th isotopologues (M*n*) generally generate up to n + 1 product ion isotopologues (if structurally possible), all of which can be off-target isotopic interfering signals. Although desirable, it is virtually impossible to manually find and resolve all possible isotopic interference in data containing hundreds of MRM channels.

To overcome this, we implemented a visual deisotoping function as one of the key features of TRACES. It first identifies all relationships of the existing MRM channels in the current dataset for possible isotopic interference. Then, using annotations linked to the MRM channels, it calculates the isotopic abundance ratio and removes interference by chromatogram subtraction. To calculate the isotopic abundance ratios, compositional formulae for MS1 (precursor ion) and MS2 (product ion or neutral loss fragment) are required, which can be inputted as optional information in the compound library. Subsequently, with a proper compound library, TRACES automatically proposes deisotoped chromatograms.

It should be noted that the deisotoping algorithm is based on channel-level annotation with putative compounds and not on compound identification. Therefore, there is a risk of inaccurate isotopic abundance ratios when the inferred compound is incorrect. Nevertheless, the major advantage of our algorithm is that it allows isotopic correction at the pretreatment step independent of peak identification.

The TRACES deisotoping algorithm considers up to M10 isotopologues for isotopic correction, which we assume is sufficient to cover all observable isotopic signals. For example, expected natural abundances of M10 isotopologues relative to M0 are 1.5 × 10^−10^ for LPC 18:0 [C_26_H_55_O_7_NP (protonated); *m/z* 524.4], 2.8 × 10^−9^ for PC 34:0 [C_42_H_85_O_8_NP (protonated); *m*/*z* 762.6], 1.1 × 10^−8^ for TG 54:3 [C_57_H_108_NO_6_ (ammonium adduct); *m*/*z* 902.8], and 8.4 × 10^−7^ for CL 72:4 (C_81_H_149_O_17_P_2_; *m*/*z* 1456.0). These M10 isotopologues are undetectable in typical TQMS with dynamic ranges less than 1.0 × 10^6^.

### 2.6. Application to Mouse Phospholipid Analysis

To validate the performance of TRACES, phospholipid analysis of mouse tissues (spleen, lung, liver, and brain) was performed as a real sample application. The tested LC-MS method contained 1412 MRM events (Supplementary Material S1) targeting headgroup-related phospholipid fragmentation. MRM transitions targeting the headgroups do not distinguish isomers of fatty chains; therefore, each chromatogram may contain multiple isomers and tends to be complex. However, this strategy is suitable for the initial profiling of unknown samples because it can cover the entire phospholipid with a relatively small number of MRM channels [15,16]. The raw data (100 files) were converted to .trc files and loaded into TRACES. A compound library for the typical phospholipid headgroup MRM (Supplementary Material S2) was loaded to annotate 949 of the 1412 channels. The entire dataset was then deisotoped.

**Figure 6:**
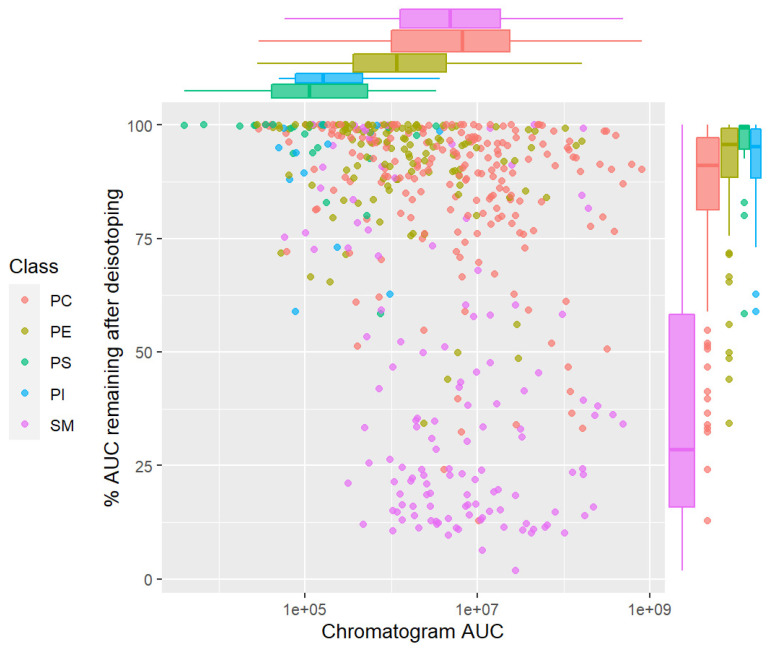
Effect of deisotoping on chromatogram area under the curve (AUC). Mouse tissue phospholipid LC-MRM-MS data were deisotoped, and mean changes in chromatogram AUC were calculated for each MRM channel using all data (100 files). Data for 486 channels with positive AUC values are plotted. Horizontal axis, chromatogram AUC before deisotoping; vertical axis, AUC after deisotoping/AUC before deisotoping (%). Boxplot shows marginal distributions.

We first evaluated the effect of deisotoping on the chromatogram area under the curve (AUC) for the MRM channels in the dataset (Supplementary Data S3 and S4). Among the 949 annotated MRM channels, 486 channels had some signal in any of the four tissue types. As shown in Figure 6, many MRM channels showed a decrease in the AUC by deisotoping to varying degrees. Signal decreases of more than 5, 10, 25 and 50% were observed in 312 (64.2%), 249 (51.2%), 148 (30.5%), and 104 (21.4%) out of 486 channels, respectively. Regarding the phospholipid class, a large decrease in AUC was found in MRM channels targeting SM, followed by those targeting PC, suggesting that chromatograms targeting these lipids contain substantial levels of isotopic signals of other phospholipids (other PCs and SMs, in this case). Chromatograms targeting phosphatidylethanolamines (PEs), phosphatidylinositols (PIs), and some PSs were also affected, albeit to a lesser magnitude.

**Figure 7:**
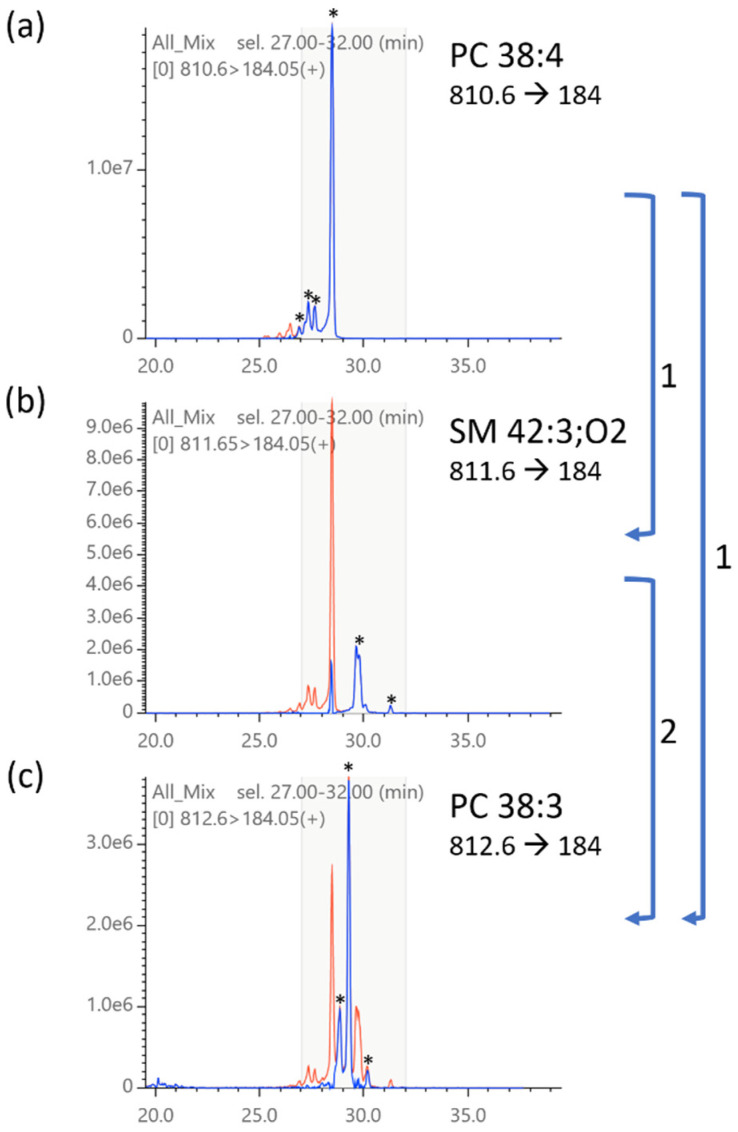
Deisotoping of MRM chromatograms. The same chromatograms shown in Figure 4 are deisotoped. Deisotoped chromatograms (blue lines) are overlaid on original chromatograms (red). TRACES performs deisotoping in the order from lower *m*/*z* to higher *m*/*z*, as shown in numbered arrows, i.e., (1) chromatogram subtraction of 810.6→184 (**a**) from 811.6→184 (**b**) and 810.6→184 (**a**) from 810.6→184 (**c**) and then (2) subtraction of 811.6→184 (**b**, deisotoped by step 1) from 812.6→184 (**c**). Asterisks indicate deduced monoisotopic peaks.

**Figure 8:**
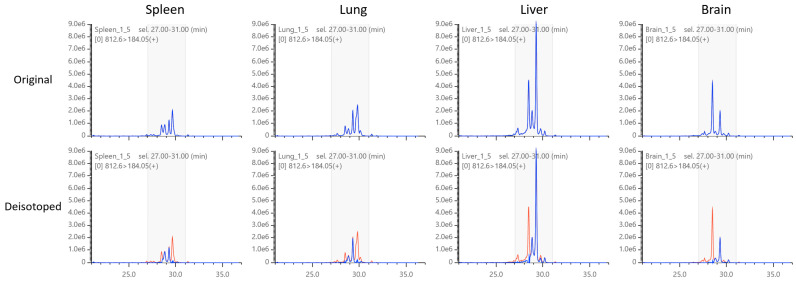
Changes in chromatographic patterns in various tissues. (**Top** panels) In original chromatograms, 4–6 peaks are observed for each tissue. Peak patterns are complex, and it is difficult to determine which peak to focus on. (**Bottom** panels) After deisotoping, only 2 major peaks and 1–2 minor peaks remained, which were easier to compare between different tissues.

Next, we examined the MRM chromatograms for changes in peak patterns by deisotoping. As shown in Figure 7, TRACES successfully removed the isotopic peaks as intended, including the nested interference of multiple MRM channels. Furthermore, we compared the peak patterns between different tissues (Figure 8). Deisotoped chromatograms showed much simpler peak patterns than the original chromatograms, which allowed us to easily compare different tissues and narrow down the peak to focus on. The results clearly demonstrate that TRACES provides a unique benefit in qualitative chromatogram analysis in addition to quantitative correction of peak areas.

**Figure 9:**
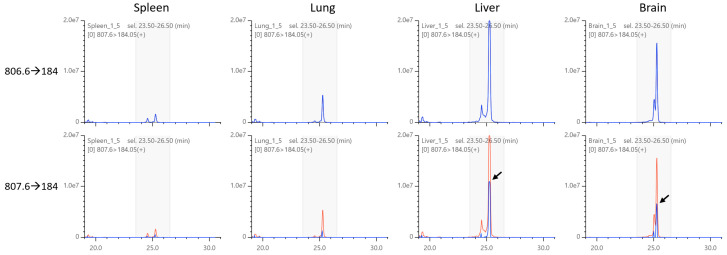
Insufficient deisotoping with saturated signals. (**Top** panes) Source chromatograms of isotopic interference. Liver and brain chromatograms are saturated or nearly saturated, respectively. (**Bottom** panels) Affected chromatograms. A significant part of signals remains after deisotoping in the liver and brain (arrows).

Although it shows satisfactory performance in various scenarios, TRACES has a limitation in deisotoping for saturated chromatograms. As shown in Figure 9, when the source chromatogram for isotopic interference has a saturating peak(s), deisotoping is insufficient around the region. Furthermore, the saturation characteristics may vary depending on instrument type; however, we did not implement any workaround for this problem. Nevertheless, chromatograms can still be used to qualitatively distinguish isotopically affected peaks. 

## 3. Materials and Methods

### 3.1. Chemicals

LCMS-grade methanol, 2-propanol, and acetonitrile were purchased from FUJIFILM Wako Pure Chemical, Osaka, Japan. Milli-Q ultrapure water (Merck Millipore, Burlington, MA, USA) was used in this study. Other chemicals of research grade were purchased from FUJIFILM Wako Pure Chemical.

### 3.2. LC-MRM-MS

A Shimadzu LCMS-8060 TQMS system (Shimadzu, Kyoto, Japan) with a Nexera UHPLC system (Shimadzu) was used for phospholipid analysis of the mouse tissue. The following parameters were used for ionization: electrospray ionization voltage, 4 kV (positive) and 3.5 kV (negative); heat block temperature, 400 °C; desolvation line temperature, 250 °C; interface temperature, 300 °C; nebulizer gas, 3 L/min; heading gas, 10 L/min; drying gas, 10 L/min. For chromatographic separation, an Acquity UPLC BEH C8 column (1.7 μm, 2.1 × 100 mm; Waters Corp, Milford, MA, USA) was used with a ternary mobile phase system (mobile phase A: 5 mM ammonium bicarbonate/water; mobile phase B: acetonitrile, mobile phase C: 2-propanol), a flow-rate of 0.35 mL/min, and column temperature of 47 °C. The following gradient time program [time (%A/%B/%C)] was used: 0 min (75/20/5)–20 min (20/75/5)–40 min (20/5/75)–45 min (5/5/90)–50 min (5/5/90)–50.1 min (75/20/5)–55 min (end)

To prepare mouse tissue lipid extracts, frozen stock tissues (~10–50 mg/sample) were crushed to powder using a cryo-mill (AutoMill; Tokken, Chiba, Japan) and extracted with 1 mL of methanol. Thereafter, methanol-insoluble fractions were collected by centrifugation (10,000× *g*, 5 min), diluted (1:10–1:500, depending on the tissue type and signal intensities), and 5 μL was injected for analysis.

A panel of phospholipid MRM transitions was prepared using the theoretical values for headgroup-related fragmentation. PC and LPC were ionized in positive mode, and the transition [M + H]^+^→184^+^ was used to monitor common product ions. For PE and LPE, [M + H]^+^→[M + H − 141]^+^ was used to monitor the common neutral loss of 141 Da. Similarly, PS/LPS and PI/LPI were monitored using [M − H]^−^→[M − H − 87]^−^ and [M − H]^−^→241^−^, respectively. A total of 1412 MRM events were set in the measurement method (Supplementary Material S1). This method has extra MRM transitions that are not covered in the compound library; most of these channels were set to check phospholipids with longer or shorter fatty chains or those with extra double bonds.

### 3.3. Software Implementation

TRACES was developed as a 64-bit Universal Windows Platform (UWP) application software using C# and XAML languages and Microsoft Visual Studio 2017. Windows 10 (build 14393) or higher is required to execute the program. The Win2D library v1.21.0 was used for chromatogram rendering [17].

The TRACES data file converter was developed as a 64-bit Windows application and requires Windows 7 with .NET Framework 4.5.2 or higher. The current version of the converter mainly supports Shimadzu TQMS .lcd files. As a vendor-neutral data format, mzML is also supported, but there is a limitation in that collision energy values may be lost from mzML files when raw data files are converted using msconvert in ProteoWizard [18], the most widely used converter. When no collision energy is found, the TRACES data file converter sets 0 V to the channel.

### 3.4. Theories for MS2-Level Isotopic Distribution and Deisotoping

Isotopic distributions for the MRM transition A→B were calculated using the composition formulae [A − B] (neutral fragment) and B (product ion). A signal for A*_i_*→B*_j_* (*i*-th and *j*-th isotopologues; *i* ≤ *j*) relative to A_0_→B_0_ (monoisotopic) can be calculated as a product of abundance ratios for [A − B]*_i–j_* and B*_j_*. TRACES refers to ‘Formula’, ‘MS2Formula’, and ‘MS2FormulaType’ fields of the compound library for calculation.

Before deisotoping, TRACES captures all ‘source MRM channel’ to ‘affected MRM channel’ relations for isotopic interference. Here, the source channels are those originally assigned to detect the target compounds, and the affected channels are those in which isotopic signals may appear. Q1 and Q3 *m*/*z* values of transitions were compared using a default mass tolerance of ±0.2 Da to determine whether the values fall within a range in which isotopic interferences may occur up to M10 isotopologues. Regarding collision energies, the current version of TRACES performs corrections only if their differences are within a ±1.0 V range since different collision energies result in different signal intensities. Thereafter, TRACES calculates the isotopic abundance ratios used as a correction factor when the source chromatograms are subtracted from the affected chromatograms. As the channel relations may be cascaded (chained), TRACES sequentially performs chromatographic subtraction starting with the channel with the lowest *m*/*z*. Over-subtraction is possible in cases where the raw data contain redundant MRM channels. To avoid this, TRACES selects the max correction factors point-by-point along the retention time axis and performs subtraction only once. For multiple annotations in source MRM channels, TRACES selects the one with the maximum correction factor by default. TRACES assumes that the data are collected using TQMS instruments calibrated at unit mass resolution. Accordingly, there is a limitation in that deisotoping may be inaccurate when the original data are collected using different mass resolutions.

### 3.5. Lipid Nomenclature and Notation

The lipid shorthand notation used in the present study is based on recent literature [19], except with a slight modification where spaces were replaced with underscores in the compound library. 

### 3.6. Data Processing and Statistics

Shimadzu LabSolutions LCMS software version 5.9 (Shimadzu) was used for instrument operation and raw data collection. The tab-delimited text data for chromatogram AUC values before and after deisotoping were exported from TRACES, and the changes were analyzed using R (version 4.1.1). 

## 4. Conclusions

In the present study, we developed a lightweight LC-MRM-MS chromatogram browser, TRACES, as a tool to facilitate the review and post-analysis of large MRM datasets. We demonstrated that not only is TRACES useful for routine review of large datasets, but it also has unique correction features that allow users to select the correct chromatographic features at the earliest stages of data processing. However, since our demonstration was presented in mouse tissue phospholipid analysis, further investigation to validate the software on other classes of compounds is necessary. We also noted that our deisotoping algorithm is limited in that the quantitative accuracy of the results is compromised when the data contain saturated signals. To overcome this problem, workarounds, such as compensation for saturated signals, may be necessary, which require further investigation. Nevertheless, we believe that this software will be useful as a tool to bridge the gap between routine small-scale manual data analysis and fully automated, complex large-scale analysis.

## Figures and Tables

**Table 1:** Field definitions of the compound library.

Field	Type	Description
Name	string	Compound name
Q1	numeric	Q1 *m*/*z* value
Q3	numeric	Q3 *m*/*z* value
CE **	numeric	Collision energy
Polarity	string	‘Positive’ or ‘Negative’
Formula *	string	Formula for precursor ion
MS2Formula *	string	Formula for product ion or neutral loss fragment
MS2FormulaTyp e *	string	‘ConstantProduct’ or ‘ConstantNeutralLoss’
Tags **	string	Any strings for search/filter

* Optional, mandatory for deisotoping; ** Optional, used in functions not detailed in this study.

**Table 2:** MS1 and MS2 isotopic distributions of PC 34:2 (theoretical).

Compound	Isotopologue	Q1 *m*/*z*	MS1 Abundance	Q3 *m*/*z*	MS2 Abundance	Affected Compound
PC 34:2 *	M0	758.6^+^	α	184^+^ **	β	(self)
	M1	759.6^+^	4.7 × 10^−1^ α	184^+^	4.1 × 10^−1^ β	SM 38:1;O2
				185^+^	6.1 × 10^−2^ β	-
	M2	760.6^+^	1.2 × 10^−1^ α	184^+^	9.0 × 10^−2^ β	PC 34:1
				185^+^	2.5 × 10^−2^ β	-
				186^+^	9.8 × 10^−3^ β	-
	M3	761.6^+^	2.4 × 10^−2^ α	184^+^	1.4 × 10^−2^ β	SM 38:0;O2
				185^+^	5.4 × 10^−3^ β	-
				186^+^	4.0 × 10^−3^ β	-
				187^+^	5.2 × 10^−4^ β	-
	M4	762.6^+^	3.7 × 10^−3^ α	184^+^	1.7 × 10^−3^ β	PC 34:0
				185^+^	8.5 × 10^−4^ β	-
				186^+^	8.8 × 10^−4^ β	-
				187^+^	2.1 × 10^−4^ β	-
				188^+^	3.8 × 10^−5^ β	-

* C_42_H_81_O_8_NP (protonated); ** C_5_H_15_O_4_NP (phosphocholine).

**Table 3:** MS1 and MS2 isotopic distributions of PS 34:2 (theoretical).

Compound	Isotopologue	Q1 *m*/*z*	MS1 Abundance	Q3 *m*/*z*	MS2 Abundance	Affected Compound
PS 34:2 *	M0	758.5^−^	α	673.5^−^ **	β	(self)
	M1	759.5^−^	4.5 × 10^−1^ α	673.5^−^	3.7 × 10^−2^ β	-
				674.5^−^	4.1 × 10^−1^ β	-
	M2	760.5^−^	1.2 × 10^−1^ α	673.5^−^	4.6 × 10^−3^ β	-
				674.5^−^	1.5 × 10^−2^ β	-
				675.5^−^	9.9 × 10^−2^ β	PS 34:1
	M3	761.5^−^	2.3 × 10^−2^ α	673.5^−^	1.6 × 10^−4^ β	-
				674.5^−^	1.9 × 10^−3^ β	-
				675.5^−^	3.7 × 10^−3^ β	-
				676.5^−^	1.7 × 10^−2^ β	-
	M4	762.5^−^	3.7 × 10^−3^ α	673.5^−^	6.3 × 10^−6^ β	-
				674.5^−^	6.3 × 10^−5^ β	-
				675.5^−^	4.5 × 10^−4^ β	-
				676.5^−^	6.5 × 10^−4^ β	-
				677.5^−^	2.5 × 10^−3^ β	PS 34:0

* C_40_H_73_O_10_NP (deprotonated); ** Product ion by neutral loss of 87 Da (C_3_H_5_O_2_N).

## Data Availability

TRACES, TRACES data file converter, and the full dataset generated in this study are available on GitHub [20]. These materials are also available via Zenodo (doi:10.5281/zenodo.6447828).

## Supplementary Materials

The following supporting information can be downloaded at: https://www.mdpi.com/article/10.3390/metabo12040354/s1, Supplementary Material S1: Scheduled MRM event list of the measurement method; Supplementary Material S2: Compound library for phospholipid headgroup MRM; Supplementary Data S3: All AUC data for the mouse tissue phospholipid analysis; Supplementary Data S4: Numerical data for Figure 6; Supplementary Material S5: TRACES program (installer) and TRACES data file converter. Supplementary Material S6: Traces data files (.trc) for the mouse tissue phospholipid analysis.

## References

[1] Kita Y., Takahashi T., Uozumi N., Shimizu T. (2005). A Multiplex Quantitation Method for Eicosanoids and Platelet-Activating Factor Using Column-Switching Reversed-Phase Liquid Chromatography–Tandem Mass Spectrometry. Anal. Biochem..

[2] Zhou J., Yin Y. (2016). Strategies for Large-Scale Targeted Metabolomics Quantification by Liquid Chromatography-Mass Spectrometry. Analyst.

[3] Luo P., Dai W., Yin P., Zeng Z., Kong H., Zhou L., Wang X., Chen S., Lu X., Xu G. (2015). Multiple Reaction Monitoring-Ion Pair Finder: A Systematic Approach To Transform Nontargeted Mode to Pseudotargeted Mode for Metabolomics Study Based on Liquid Chromatography–Mass Spectrometry. Anal. Chem..

[4] Pino L.K., Searle B.C., Bollinger J.G., Nunn B., MacLean B., MacCoss M.J. (2020). The Skyline Ecosystem: Informatics for Quantitative Mass Spectrometry Proteomics. Mass Spectrom. Rev..

[5] Reiter L., Rinner O., Picotti P., Hüttenhain R., Beck M., Brusniak M.-Y., Hengartner M.O., Aebersold R. (2011). MProphet: Automated Data Processing and Statistical Validation for Large-Scale SRM Experiments. Nat. Methods.

[6] Nasso S., Goetze S., Martens L. (2015). Ariadne’s Thread: A Robust Software Solution Leading to Automated Absolute and Relative Quantification of SRM Data. J. Proteome Res..

[7] Tsugawa H., Kanazawa M., Ogiwara A., Arita M. (2014). MRMPROBS Suite for Metabolomics Using Large-Scale MRM Assays. Bioinformatics.

[8] Tsugawa H., Ohta E., Izumi Y., Ogiwara A., Yukihira D., Bamba T., Fukusaki E., Arita M. (2015). MRM-DIFF: Data Processing Strategy for Differential Analysis in Large Scale MRM-Based Lipidomics Studies. Front. Genet..

[9] Adams K.J., Pratt B., Bose N., Dubois L.G., St. John-Williams L., Perrott K.M., Ky K., Kapahi P., Sharma V., MacCoss M.J. (2020). Skyline for Small Molecules: A Unifying Software Package for Quantitative Metabolomics. J. Proteome Res..

[10] Chitpin J.G., Surendra A., Nguyen T.T., Taylor G.P., Xu H., Alecu I., Ortega R., Tomlinson J.J., Crawley A.M., McGuinty M. (2022). BATL: Bayesian Annotations for Targeted Lipidomics. Bioinformatics.

[11] Gao L., Ji S., Burla B., Wenk M.R., Torta F., Cazenave-Gassiot A. (2021). LICAR: An Application for Isotopic Correction of Targeted Lipidomic Data Acquired with Class-Based Chromatographic Separations Using Multiple Reaction Monitoring. Anal. Chem..

[12] Han X., Gross R.W. (2005). Shotgun Lipidomics: Electrospray Ionization Mass Spectrometric Analysis and Quantitation of Cellular Lipidomes Directly from Crude Extracts of Biological Samples. Mass Spectrom. Rev..

[13] Liebisch G., Lieser B., Rathenberg J., Drobnik W., Schmitz G. (2004). High-Throughput Quantification of Phosphatidylcholine and Sphingomyelin by Electrospray Ionization Tandem Mass Spectrometry Coupled with Isotope Correction Algorithm. Biochim. Biophys. Acta (BBA) Mol. Cell Biol. Lipids.

[14] Castellaneta A., Losito I., Coniglio D., Leoni B., Santamaria P., di Noia M.A., Palmieri L., Calvano C.D., Cataldi T.R.I. (2021). *LIPIC*: An Automated Workflow to Account for Isotopologue-Related Interferences in Electrospray Ionization High-Resolution Mass Spectra of Phospholipids. J. Am. Soc. Mass Spectrom..

[15] Hashidate-Yoshida T., Harayama T., Hishikawa D., Morimoto R., Hamano F., Tokuoka S.M., Eto M., Tamura-Nakano M., Yanobu-Takanashi R., Mukumoto Y. (2015). Fatty Acid Remodeling by LPCAT3 Enriches Arachidonate in Phospholipid Membranes and Regulates Triglyceride Transport. eLife.

[16] Valentine W.J., Mostafa S.A., Tokuoka S.M., Hamano F., Inagaki N.F., Nordin J.Z., Motohashi N., Kita Y., Aoki Y., Shimizu T. (2022). Lipidomic Analyses Reveal Specific Alterations of Phosphatidylcholine in Dystrophic Mdx Muscle. Front. Physiol..

[17] Win2D. https://github.com/microsoft/Win2D.

[18] Chambers M.C., Maclean B., Burke R., Amodei D., Ruderman D.L., Neumann S., Gatto L., Fischer B., Pratt B., Egertson J. (2012). A Cross-Platform Toolkit for Mass Spectrometry and Proteomics. Nat. Biotechnol..

[19] Liebisch G., Fahy E., Aoki J., Dennis E.A., Durand T., Ejsing C.S., Fedorova M., Feussner I., Griffiths W.J., Köfeler H. (2020). Update on LIPID MAPS Classification, Nomenclature, and Shorthand Notation for MS-Derived Lipid Structures. J. Lipid Res..

[20] TRACES. https://github.com/KitaYoshihiro/TRACES/releases/tag/v0.2.48.0-update.1.

